# Antibody responses to BNT162b2 SARS‐CoV‐2 mRNA vaccine among healthcare workers and residents of long‐term care facilities: A cohort study in Northern Italy

**DOI:** 10.1002/hsr2.1087

**Published:** 2023-02-11

**Authors:** Costanza Vicentini, Carla Maria Zotti, Alessandro Roberto Cornio, Jacopo Garlasco, Noemi Marengo, Davide Meddis, Savina Ditommaso, Monica Giacomuzzi, Gabriele Memoli, Valerio Bordino, Maria Michela Gianino, the Collaborating Group

**Affiliations:** ^1^ Department of Public Health and Paediatrics University of Turin Turin Italy

**Keywords:** immune senescence, Italy, nursing homes, Pfizer/BNT162b2, SARS‐CoV‐2

## Abstract

**Background and Aims:**

Long‐term care facilities (LTCFs) have been severely impacted by COVID‐19, with a disproportionate amount of SARS‐CoV‐2 infections and related deaths occurring among residents.

**Methods:**

This study is part of an ongoing multicenter, prospective cohort study conducted among healthcare workers (HCWs) and residents of 13 LTCFs in Northern Italy designed to evaluate SARS‐CoV‐2 specific immunoglobulin class G (IgG) titers before and following vaccination with Pfizer/BNT162b2 SARS‐CoV‐2 mRNA vaccine (two doses of vaccine, 21 days apart). Serum samples were obtained from participants (t0) before vaccination, and (t1) 2 weeks after and analyzed to determine anti‐S1 IgG antibodies.

**Results:**

Five hundred and thirty‐four participants were enrolled (404 subjects participated in both blood draws). Seropositivity was 50.19% at t0 and 99% at t1, with a significant difference in IgG titers. A higher proportion of residents were seropositive at t0 compared with HCWs, with significantly higher IgG titers among residents at both t0 and t1. Pre‐existing immunity also had a significant effect on postvaccination IgG titers. However, a significant difference in titers at t1 between HCWs and residents considering only participants seropositive at t0 was found, with higher median titers among previously seropositive residents.

**Conclusion:**

Findings of this study provide scientific evidence endorsing the policy of universal vaccination in LTCFs.

## 1 INTRODUCTION

Long‐term care facilities (LTCFs) residents represent a high‐risk population in a high‐risk setting for SARS‐CoV‐2 transmission.^1^ LTCFs have been severely impacted by COVID‐19, with a disproportionate amount of SARS‐CoV‐2 infections and related deaths occurring among residents in several countries, and in Northern Italy in particular.^2^, ^3^ Based on Italian data, the ratio of COVID‐related mortality comparing LTCFs residents and people aged over 70 living in the community was estimated to be 3:1.^3^

Multiple vaccines against COVID‐19 were developed at unprecedented speed, with new vaccine modalities such as mRNA‐based vaccines receiving emergency approval.^4^, ^5^ Clinical trials report these vaccines are both safe and effective.^6^ In Italy, the vaccination campaign against SARS‐CoV‐2 began in December 2020, with the immunization offered initially to priority groups including healthcare workers (HCWs) and residents of LTCFs. By September 2021, over 90% of LTCF residents had completed a full vaccination cycle. The incidence of weekly SARS‐CoV‐2 infections among LTCF residents in Italy sharply decreased since the introduction of the vaccine, dropping from around 3.2% new cases per week in November 2020 to 0.01% new cases per week in May–June 2021. The percentage of deceased SARS‐CoV‐2‐positive residents among all LTCF residents also saw an important reduction, and was lower than 0.01% in mid‐September 2021.^7^

However, clinical trial data on post‐vaccine response among elderly and frail individuals is limited. Adaptive immunosenescence, a phenomenon tied to age‐related declining immune efficiency, could affect the response to SARS‐CoV‐2 vaccinations.^4^, ^8^, ^9^, ^10^ This issue is important as older adults are the population at higher risk of developing severe COVID‐19.^11^, ^12^, ^13^, ^14^

The purpose of this multicentric study was to describe the antibody response to Pfizer/BNT162b2 SARS‐CoV‐2 mRNA vaccine among individuals at high risk of exposure due to the environment in which they live or work: residents and HCWs of LTCFs. We aimed to provide real‐world data from populations, which may have been underrepresented in clinical trials.^2^

## 2 METHODS

### 2.1 Study design and participants

This study is part of an ongoing multicenter, prospective cohort study conducted among HCWs (Physicians, Nurses, and Ancillary staff) and residents of 13 LTCFs of the region of Piedmont, in Northern Italy, designed to evaluate SARS‐CoV‐2 specific IgG titers before and following a complete vaccination cycle with Pfizer/BNT162b2 SARS‐CoV‐2 mRNA vaccine (two doses of vaccine, 21 days apart).^15^ Participants were recruited on a voluntary basis in January 2021, and completed the vaccination cycle between January and March 2021. The study was approved by relevant institutional review boards (protocol numbers COV 28/2020, 10077, and 0016945), and was conducted in accordance with the Declaration of Helsinki and fulfilled the requirements of Italian (Law 2003/196) and European regulations (GDPR EC/2016/679) concerning data protection and privacy.

### 2.2 Data collection

Serum samples were obtained from participants at two‐time points: (t0) before vaccination, and (t1) 2 weeks after completing a full vaccination cycle. Specimens were processed for cryopreservation as previously described.^15^ Demographic characteristics of enrolled subjects, as well as information concerning previous SARS‐CoV‐2 infections confirmed by reverse‐transcription polymerase chain reaction (RT‐PCR) testing, were collected from the Health Directorates of the involved facilities and checked on the regional database in which all official swabs are registered. Further, participants were asked whether they had previously been infected by SARS‐CoV‐2 and if so, when.

In compliance to regional guidelines, from October 2020 all staff and residents of LTCFs are screened for SARS‐CoV‐2 on a biweekly basis, regardless of symptoms related to COVID‐19. Informed consent was obtained before collection of data and specimens.

### 2.3 Laboratory analysis

The analysis was performed at the Laboratory of Serology and Microbiology applied to Hygiene of the Department of Public Health and Paediatrics of the University of Turin. SARS‐CoV‐2 immunoglobulin class G (IgG) antibodies were assayed using the EUROIMMUN QuantiVac ELISA kit (EUROIMMUN Medizinische Labordiagnostika AG), as previously described.^15^ This method detects IgG antibodies using the S1 domain of the spike protein including the receptor‐binding domain. Results were expressed in relative units, RU/mL as follows: negative if <8 RU/mL, borderline if between 8 and 11 RU/mL, and positive if ≥11 RU/mL. A conversion factor of 3.2 was identified by the manufacturer to convert RU/mL into binding antibody units (BAU/mL).

### 2.4 Statistical analysis

Demographic and clinical characteristics, including IgG‐S titers, were summarized using descriptive statistics. Medians and interquartile ranges (IQRs) were used to describe continuous variables, due to nonnormal distribution (Shapiro–Wilk test), and categorical variables were reported as numbers and percentages. Statistically significant differences in categorical and continuous variables were investigated using *χ* ^2^ and Mann–Whitney *U* tests, respectively.

The Wilcoxon signed‐rank test was performed to compare IgG measurements between the first and second blood draws (t0 vs. t1). Mann–Whitney *U* tests were conducted to evaluate differences in antibody titers at t1 among HCWs versus residents of LTCFs, and between subjects with and without a previous SARS‐CoV‐2 infection confirmed by a positive RT‐PCR test. The significance level for all analyses was set at two‐tailed 0.05. All analyses were conducted using SPSS version 27.0 (IBM).

## 3 RESULTS

**Figure 1:**
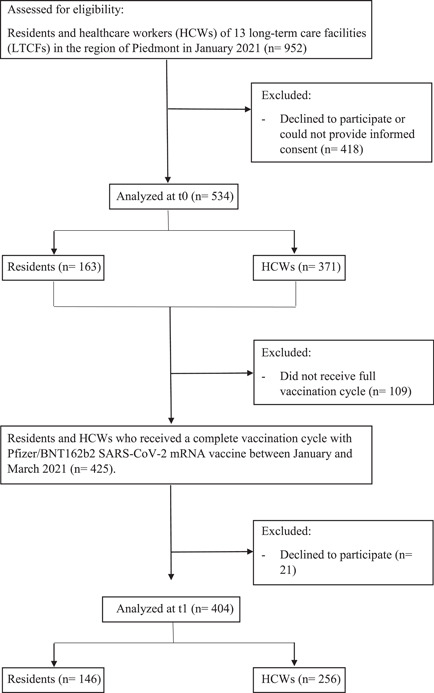
Study flowchart. Serum samples were obtained from participants at two timepoints: (t0) before vaccination, and (t1) 2 weeks after completing a full vaccination cycle (two doses of vaccine, 21 days apart).

Among 952 eligible HCWs and residents of 13 LTCFs, 534 participants were enrolled, and 404 subjects participated in both blood draws. A flowchart of study participants is presented in Figure 1. Table 1 reports demographic and clinical characteristics of participants, stratified according to subject type. HCWs were significantly younger and more often female compared to residents. A significantly higher proportion of residents had a previous SARS‐CoV‐2 infection confirmed by RT‐PCR.

**Table 1:** Demographic and clinical characteristics of healthcare workers (HCWs) and residents of 13 long‐term care facilities (LTCFs) of the region of Piedmont, in Northern Italy, January 2021 (*n* = 534).

Characteristic	HCWs (*n* = 371)	Residents (*n* = 163)	*p* value^a^
Age at enrollment, median (IQR), years	47 (38–54)	86 (90–80)	<0.001
Female gender, *N* (%)	308 (83.01)	115 (70.55)	<0.001
Previous SARS‐CoV‐2 infection confirmed by RT‐PCR, *N* (%)	175 (47.17)	106 (65.03)	<0.001
Days between last positive RT‐PCR and 2nd blood draw, median (IQR)^b^	109 (92–262)	99 (72–302)	0.146

Abbreviations: IQR, interquartile range; RT‐PCR, reverse‐transcription polymerase chain reaction.

^a^ Differences in categorical and continuous variables were investigated using *χ* ^2^ and Mann–Whitney *U* tests, respectively.

^b^ Among subjects with a previous SARS‐CoV‐2 infection confirmed by RT‐PCR.

Seropositivity among all participants was 50.19% at t0 and 99% at t1. The median IgG titer was 11.34 RU/mL (IQR 0–47.43 RU/mL) at t0 and 1497.28 RU/mL (IQR 779.57– 2698.24 RU/mL) at t1 among all subjects. A significant difference in IgG titers at t0 versus t1 was found (*p* < 0.001 at Wilcoxon signed‐rank test).

**Figure 2:**
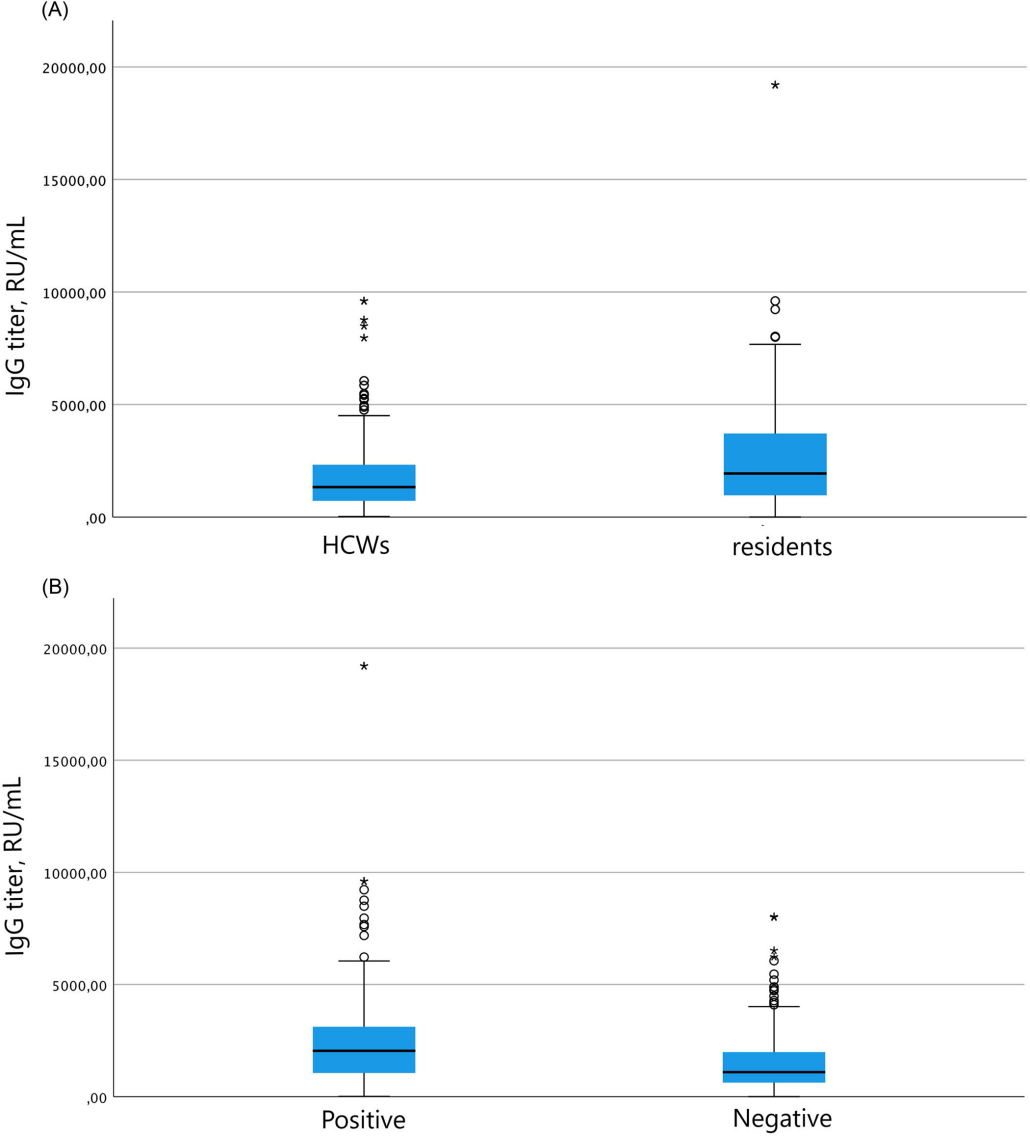
Immunoglobulin class G (IgG) titers 2 weeks after receiving two doses of Pfizer/BNT162b2 SARS‐CoV‐2 mRNA vaccine among healthcare workers (HCWs) and residents of 13 long‐term care facilities (LTCFs) of the region of Piedmont, in Northern Italy, January–March 2021 (*n* = 404). (A) According to subject type (HCWs vs. residents). (B) According to previous SARS‐CoV‐2 infection confirmed by reverse‐transcription polymerase chain reaction (RT‐PCR). Circles indicate the outliers and asterisks extreme outliers.

Table 2a,b reports IgG titers among HCWs and residents at t0 and t1, among all participants (Table 2a) and among participants seropositive at t0 (Table 2b). Significant differences were found comparing HCWs and residents at both time points. A higher proportion of residents were seropositive at t0 compared with HCWs, with significantly higher IgG titers among residents, both considering all included participants and only participants seropositive at t0. After vaccination, 100% of HCWs were seropositive whereas there were two nonresponders among residents. Nonresponding individuals were both males, aged 82 and 87 years, and both had no previous SARS‐CoV‐2 infection confirmed by RT‐PCR. Residents had significantly higher titers compared with HCWs at t1 (Figure 2A).

**Table 2a:** Differences in immunoglobulin class G (IgG) titers (t0) before and (t1) 2 weeks after receiving two doses of Pfizer/BNT162b2 SARS‐CoV‐2 mRNA vaccine between healthcare workers (HCWs) and residents of 13 long‐term care facilities (LTCFs) of the region of Piedmont, in Northern Italy, January–March 2021 (including all study participants: *n* = 534 and 404).

	HCWs	Residents	*p* value^a^
Draw 1 (t0)			
Positive for SARS‐CoV‐2 IgG, *N* (%)	162 (43.66)	106 (65.03)	
Median titer (IQR), RU/mL	6.23 (0–34.29)	22.97 (5.16–78.85)	<0.001
Draw 2 (t1)			
Positive for SARS‐CoV‐2 IgG, *N* (%)	256 (100)	144 (98.63)	
Median titer (IQR), RU/mL	1333.36 (727.72–2323.60)	1992.96 (992.91−4002.00)	0.002

Abbreviation: IQR, interquartile range.

^a^ Difference in IgG titers assessed with Mann–Whitney *U* test.

**Table 2b:** Differences in immunoglobulin class G (IgG) titers (t0) before and (t1) 2 weeks after receiving two doses of Pfizer/BNT162b2 SARS‐CoV‐2 mRNA vaccine between healthcare workers (HCWs) and residents of 13 long‐term care facilities (LTCFs) of the region of Piedmont, in Northern Italy, January–March 2021 (including only participants seropositive at t0: *n* = 268 and *n* = 201).

	HCWs	Residents	*p* value^a^
Draw 1 (t0)			
Positive for SARS‐CoV‐2 IgG, *N* (%)	162 (100)	106 (100)	
Median titer (IQR), RU/mL	39.27 (22.97–85.71)	63.34 (25.33–120.81)	0.016
Draw 2 (t1)			
Positive for SARS‐CoV‐2 IgG, *N* (%)	110 (100)	91 (100)	
Median titer (IQR), RU/mL	2142.16 (1224.72–3169.7)	2533.5 (1286.1–4662.24)	0.044

^a^ Difference in IgG titers assessed with Mann–Whitney *U* test.

As shown in Table 3, significant differences were also found at both time points comparing subjects stratified according to previous SARS‐CoV‐2 infection confirmed by RT‐PCR. At t0, a higher proportion of participants with a previous infection were seropositive compared with participants without a previous infection. IgG titers were significantly higher at t0 in previously infected participants. After vaccination, seropositivity in both groups neared 99%, although median IgG titers were almost doubled in previously infected subjects (Figure 2B). However, as summarized in Table 4, when stratifying IgG titers among HCWs and residents according to previous SARS‐CoV‐2 infection confirmed by RT‐PCR testing, only the difference in titers at t0 between HCWs and residents without a previous SARS‐CoV‐2 infection confirmed by RT‐PCR testing maintained statistical significance.

**Table 3:** Differences in immunoglobulin class G (IgG) titers (t0) before and (t1) 2 weeks after receiving two doses of Pfizer/BNT162b2 SARS‐CoV‐2 mRNA vaccine among healthcare workers (HCWs) and residents of 13 long‐term care facilities (LTCFs) of the region of Piedmont, in Northern Italy, stratified according to previous SARS‐CoV‐2 infection confirmed by RT‐PCR, January–March 2021 (*n* = 534 and 404).

	Previous SARS‐CoV‐2 infection confirmed by RT‐PCR	No previous SARS‐CoV‐2 infection confirmed by RT‐PCR	*p* value^a^
Draw 1 (t0)			
Positive for SARS‐CoV‐2 IgG, *N* (%)	212 (75.44)	56 (22.13)	
Median titer (IQR), RU/mL	29.94 (11.53–75.61)	0 (0–9.59)	<0.001
Draw 2 (t1)			
Positive for SARS‐CoV‐2 IgG, *N* (%)	206 (100)	194 (99)	
Median titer (IQR), RU/mL	2062.72 (1063.01–3152.40)	1104.57 (628.24–2019.76)	<0.001

Abbreviations: IQR, interquartile range; RT‐PCR, reverse‐transcription polymerase chain reaction.

^a^ Difference in IgG titers assessed with Mann–Whitney *U* test.

**Table 4:** Differences in immunoglobulin class G (IgG) titers (t0) before and (t1) 2 weeks after receiving two doses of Pfizer/BNT162b2 SARS‐CoV‐2 mRNA vaccine among healthcare workers (HCWs) and residents of 13 long‐term care facilities (LTCFs) of the region of Piedmont, in Northern Italy, stratified according to previous SARS‐CoV‐2 infection confirmed by RT‐PCR testing, January–March 2021 (*n* = 534 and 404).

	HCWs		Residents		*p* value^a^	
	(a) Previous SARS‐CoV‐2 infection confirmed by RT‐PCR	(b) No previous SARS‐CoV‐2 infection confirmed by RT‐PCR	(c) Previous SARS‐CoV‐2 infection confirmed by RT‐PCR	(d) No previous SARS‐CoV‐2 infection confirmed by RT‐PCR	(a) versus (c)	(b) versus (d)
Draw 1 (t0)						
Positive for SARS‐CoV‐2 IgG, *N* (%)	131 (24.53)	31 (5.81)	81 (15.17)	25 (4.68)		
Median titer (IQR), RU/mL	26.52 (10.83–58.42)	0.00 (0.00–3.39)	41.71 (11.51–105.14)	8.19 (0.00–48.07)	0.141	<0.001
Draw 2 (t1)						
Positive for SARS‐CoV‐2 IgG, *N* (%)	114 (28.22)	142 (35.15)	92 (22.77)	52 (12.87)		
Median titer (IQR), RU/mL	1905.16 (940.44–2911.22)	971.96 (650.95–1888.98)	2279.32 (1158.14–4002.06)	1345.6 (482.39–3982.56)	0.063	0.32

Abbreviations: IQR, interquartile range; RT‐PCR, reverse‐transcription polymerase chain reaction.

^a^ Difference in IgG titers assessed with Mann–Whitney *U* test.

## 4 DISCUSSION

This cohort study conducted among HCWs and residents of 13 LTCFs in Northern Italy found IgG titers 2 weeks after a full vaccination cycle were significantly upregulated compared with before vaccination among all participants. Our results add to an emerging literature supporting the real‐life effectiveness of mRNA vaccines in inducing detectable antibody responses in this setting.^2^, ^13^, ^16^, ^17^, ^18^, ^19^

Following a complete vaccination cycle, 100% of HCWs were seropositive, whereas there were two nonresponders among residents. This finding highlights the importance of maintaining infection control practices and nonpharmaceutical interventions in LTCFs even after comprehensive vaccination campaigns to safeguard nonresponders.

Older adults are at increased susceptibility to infections due to immune‐senescence, which may also lead to decreased effectiveness of immunizations.^8^, ^10^ Age alone is likely not the sole contributor to this phenomenon: among other factors, functional decline (or frailty syndrome) has also been associated with impaired responses to vaccinations.^20^, ^21^ Concerning COVID‐19 in particular, previous investigations have found that people aged 65 years or older had lower protection against SARS‐CoV‐2 reinfection compared with younger individuals,^22^ and that the antibody response induced by a single dose of Pfizer/BNT162b2 SARS‐CoV‐2 mRNA vaccine was inversely proportional to age.^23^, ^24^ Multiple comorbidities have also been correlated with reduced antibody responses following SARS‐CoV‐2 vaccination.^23^, ^24^ This study found a significant difference in median IgG titers comparing HCWs and residents, with higher titers among residents at both blood draws. Contrary to our results, other studies have found significantly lower IgG responses following a full vaccination cycle with Pfizer/BNT162b2 among elderly compared with younger individuals.^4^, ^5^ However, these studies did not specifically compare vaccine‐induced titers among LTCF residents and HCWs, but younger versus older vaccinees.^4^, ^5^ Further, Reber et al.^10^ have suggested that antibody responses in elderly individuals may be affected by qualitative more than quantitative changes, therefore the higher titers found among residents in our study might not translate into a lower infection risk. Longer‐term studies are needed to investigate this relationship, particularly in light of emerging evidence of infections in vaccinated individuals.

Recent evidence suggests pre‐existing immunity to SARS‐CoV‐2 plays an important role in determining postvaccination immunity.^24^, ^25^, ^26^ In their study of French nursing home residents, Blain et al.^25^ found higher median IgG titers following one dose of Pfizer/BNT162b2 among previously infected participants than after two doses among COVID‐naïve residents. In our study, previous SARS‐CoV‐2 exposure was also identified as an important determinant of postvaccination immunity, with titers among previously infected participants nearly double those of participants without a previous positive RT‐PCR test. Further, a significantly higher proportion of residents had a previous SARS‐CoV‐2 infection confirmed by RT‐PCR compared with HCWs, which could explain the higher titers among residents found in this study.

LTCFs represent high‐risk congregate settings for viral transmission, where residents are prevalently elder and frail.^27^, ^28^ Elder age has been associated with increased COVID‐19 severity and duration, higher peak viral loads, and delayed viral clearance.^29^, ^30^ A cohort study conducted in 100 LTCFs in the United Kingdom found more than 80% of samples obtained from polymerase chain reaction (PCR) testing of primary infections of HCWs and residents had cycle threshold (Ct) values lower than 30, which are associated with higher viral loads, and that median reinfection Ct values were lower in residents compared to HCWs, indicating higher viral loads among residents.^8^ Cascading superspreading events have a high potential of occurring in LTCFs, with a concentration of highly infectious cases among highly susceptible individuals. LTCF residents represent a population at higher risk of repeated exposure to SARS‐CoV‐2,^28^, ^31^ even compared with staff.^31^ The natural boosting of antibodies due to continued intrafacility transmission could explain results of the current study and our previous findings suggesting a more durable antibody response over time found in residents compared to HCWs of the same LTCFs.^15^

This study had limitations that could affect the generalizability of our findings. First, our cohort consisted of voluntary participants, which may have determined selection bias, and was limited by sample size. Residents lacking the capacity to consent or receiving end‐of‐care life were less likely to participate than healthier residents, and other unmeasured differences between participants and individuals declining to participate cannot be excluded. However, we were able to recruit 13 LTCFs varying in size, resident characteristics, and type of care provided. Second, our analysis was restricted by absence of data concerning the timing and clinical characteristics of previous infections, underlying conditions, and frailty status of participants. Further, even though the same regional screening protocol for SARS‐CoV‐2 RT‐PCR testing was applied in all participating LTCFs since 2020, we cannot exclude that some infections were undiagnosed, as appears to be suggested by results presented in Table 3. Previous analyses suggest surveillance data from the initial stages of the pandemic in Northern Italy were affected by underascertainment.^32^ Further, we could not account for other explanations than undetected prior infection for the low‐level IgG seen in some participants with no known prior infection, such as cross‐reactivity. Finally, it must also be noted that, as previously discussed, seropositivity may not correlate with protection against reinfection; it is known that the antibodies are only a part of the immune machine, as the role of memory T cells in killing virus‐infected cells is equally fundamental.^8^, ^13^

It remains to be determined whether infection risk will be different among vaccinated residents and HCWs, and among previously infected versus naïve individuals.

In conclusion, notwithstanding these limitations, our results support the effectiveness of the Pfizer/BNT162b2 mRNA vaccine in inducing detectable antibody responses among residents and HCWs of LTCFs. Findings of this study provide scientific evidence endorsing the policy of universal vaccination in this setting, and suggest SARS‐CoV‐2 surveillance and adherence with current infection control recommendations should be maintained. In this study, being a resident and pre‐existing immunity had a significant effect on postvaccination IgG titers. In the absence of definitive data on antibody titers correlating with protection, future studies are needed to follow the dynamics of antibody response over time, and to further investigate the relationship between age, frailty, previous infection, and antibody response.

## AUTHOR CONTRIBUTIONS

**Costanza Vicentini**: Conceptualization; supervision; writing – original draft. **Carla Maria Zotti**: Conceptualization; supervision; validation; writing – review and editing. **Alessandro Roberto Cornio**: Data curation; visualization. **Jacopo Garlasco**: Formal analysis; methodology; validation. **Noemi Marengo**: Investigation; methodology. **Davide Meddis**: Data curation; investigation; methodology; software. **Savina Ditommaso**: Investigation; methodology. **Monica Giacomuzzi**: Investigation; methodology; validation; visualization. **Gabriele Memoli**: Data curation; formal analysis; methodology. **Valerio Bordino**: Conceptualization; formal analysis; investigation; writing – review and editing. **Maria Michela Gianino**: Supervision; writing – review and editing.

## CONFLICT OF INTEREST STATEMENT

The authors declare no conflict of interest.

## ETHICS STATEMENT

The research protocol was in accordance with the Declaration of Helsinki and fulfilled the requirements of Italian (Law 2003/196) and European regulations (GDPR EC/2016/679) concerning data protection and privacy. All study procedures were reviewed and approved by the Ethical Boards of the relevant institutions (Local Health Authorities of Alessandria, Cuneo, and Turin) and the approval was subsequently confirmed by the Ethical Board of the University of Turin (protocol numbers COV 28/2020, 10077 and 0016945).

## TRANSPARENCY STATEMENT

The lead author Valerio Bordino affirms that this manuscript is an honest, accurate, and transparent account of the study being reported; that no important aspects of the study have been omitted; and that any discrepancies from the study as planned (and, if relevant, registered) have been explained.

## Data Availability

Data will be made available upon reasonable request.
